# High-resolution characterization of short-term temporal variability in the taxonomic and resistome composition of wastewater influent

**DOI:** 10.1099/mgen.0.000983

**Published:** 2023-05-05

**Authors:** Kevin K. Chau, T. Goodall, M. Bowes, K. Easterbrook, H. Brett, J. Hughes, D.W. Crook, D.S. Read, A.S. Walker, N. Stoesser

**Affiliations:** ^1^​ Nuffield Department of Medicine, University of Oxford, John Radcliffe Hospital, Headley Way, Headington, Oxford, OX3 9DU, UK; ^2^ NIHR Health Protection Research Unit in Healthcare Associated Infections and Antimicrobial Resistance, University of Oxford in partnership with Public Health England, Oxford, UK; ^3^​ UK Centre for Ecology & Hydrology, MacLean Bldg, Benson Ln, Crowmarsh Gifford, Wallingford, OX10 8BB, UK; ^4^​ Thames Water, Clearwater Court, Vastern Road, Reading, RG1 8DB, UK; ^5^​ Department of Microbiology/Infectious diseases, Oxford University Hospitals NHS Foundation Trust, John Radcliffe Hospital, Headley Way, Headington, Oxford, OX3 9DU, UK; ^6^​ NIHR Oxford Biomedical Research Centre, The Joint Research Office, Second Floor, OUH Cowley, Unipart House Business Centre, Garsington Road, Oxford, OX4 2PG, UK

**Keywords:** wastewater-based epidemiology, antimicrobial resistance, wastewater sampling, 16S rRNA, resistome, metagenomics

## Abstract

Wastewater-based epidemiology (WBE) for population-level surveillance of antimicrobial resistance (AMR) is gaining significant traction, but the impact of wastewater sampling methods on results is unclear. In this study, we characterized taxonomic and resistome differences between single-timepoint-grab and 24 h composites of wastewater influent from a large UK-based wastewater treatment work [WWTW (population equivalent: 223 435)]. We autosampled hourly influent grab samples (*n*=72) over three consecutive weekdays, and prepared additional 24 h composites (*n*=3) from respective grabs. For taxonomic profiling, metagenomic DNA was extracted from all samples and 16S rRNA gene sequencing was performed. One composite and six grabs from day 1 underwent metagenomic sequencing for metagenomic dissimilarity estimation and resistome profiling. Taxonomic abundances of phyla varied significantly across hourly grab samples but followed a repeating diurnal pattern for all 3 days. Hierarchical clustering grouped grab samples into four time periods dissimilar in both 16S rRNA gene-based profiles and metagenomic distances. 24H-composites resembled mean daily phyla abundances and showed low variability of taxonomic profiles. Of the 122 AMR gene families (AGFs) identified across all day 1 samples, single grab samples identified a median of six (IQR: 5–8) AGFs not seen in the composite. However, 36/36 of these hits were at lateral coverage <0.5 (median: 0.19; interquartile range: 0.16–0.22) and potential false positives. Conversely, the 24H-composite identified three AGFs not seen in any grab with higher lateral coverage (0.82; 0.55–0.84). Additionally, several clinically significant human AGFs (*bla*_VIM_, *bla*_IMP_, *bla*_KPC_) were intermittently or completely missed by grab sampling but captured by the 24 h composite. Wastewater influent undergoes significant taxonomic and resistome changes on short timescales potentially affecting interpretation of results based on sampling strategy. Grab samples are more convenient and potentially capture low-prevalence/transient targets but are less comprehensive and temporally variable. Therefore, we recommend 24H-composite sampling where feasible. Further validation and optimization of WBE methods is vital for its development into a robust AMR surveillance approach.

## Data Summary

All supporting data and protocols have been provided within the article or as supplementary data files. Raw sequencing data are available under ENA project PRJEB52722 (https://www.ebi.ac.uk/ena/browser/view/PRJEB52722) and PRJEB54594 (https://www.ebi.ac.uk/ena/browser/view/PRJEB54594), with all metadata included in File S1, available with the online version of this article. R scripts and markdown renders are available at https://github.com/KaibondChau/Chau_etal_Hires.

**Impact Statement**

Wastewater-based epidemiology (WBE) is an increasingly promising surveillance approach for monitoring population-level antimicrobial resistance (AMR), due to its potential for monitoring large populations at scale with relative ease. Wastewater represents a pooled sample which reflects the source population, and can be analysed to generate rich population-representative datasets with reduced logistical or ethical barriers compared to large-scale individual-level sampling. Current AMR surveillance systems typically rely on data generated through clinical microbiology laboratories, limiting most data to phenotypic results for specific pathogens. This narrow clinical snapshot hampers the surveillance of high-risk AMR-associated clones and specific AMR-associated genetic determinants which are frequently disseminated ‘silently’ through colonization rather than infection, whilst inherently biasing surveillance efforts towards healthcare-associated populations. Wastewater may act as a convergence point of both healthcare- and community-associated populations, thus potentially avoiding selection bias and capturing AMR prevalence of commensal organisms in healthy individuals, thought to constitute a major proportion of the true AMR burden. Most previous studies exploring WBE for population-level AMR surveillance have relied on single-volume grab sampling for the collection of wastewater – probably due to the convenience and significantly lower workload/cost compared to more comprehensive methods. However, the composition of wastewater is likely to fluctuate on extremely short timescales due to human behaviours. Naturally, this can be expected to have implications for WBE, but prior to the work presented here, the impact of grab versus composite sampling on results has not been assessed. Here, we comprehensively characterized the short-term temporal fluctuations in wastewater taxonomy and resistome in the context of sampling methodologies, their impact on downstream analyses and use-cases for WBE-based AMR surveillance.

## Introduction

Antimicrobial resistance (AMR) represents a significant challenge to global health [1]. Surveillance efforts are essential to monitor trends, identify emergence and develop interventions [2]. Wastewater-based epidemiology (WBE) is of increasing interest as a convenient surveillance approach, leveraging the pooling of human excreta to generate information on human populations at scale [3]. WBE is a method of studying population health by analysing the contents of wastewater/sewage for indicators of disease and substance use. Recent AMR-focused WBE studies have surveyed global AMR gene distributions [4], identified associations between AMR genes in clinical and wastewater samples [5, 6], and characterized the relationship between wastewater AMR abundance, antimicrobial usage and clinical isolate phenotype [7]. In addition to being more convenient than individual sampling for population-level surveillance, WBE can circumvent selection biases such as the typical healthcare-associated focus of most current surveillance efforts [8], by simultaneously capturing healthcare- and community-associated populations, and reflecting carried organisms thought to silently comprise most of the true AMR burden [9]. Traditional syndromic surveillance is limited as most AMR genes are carried and exchanged asymptomatically within human or other niches as part of bacterial interactions in the microbiome [10]. AMR is a suitable candidate for wastewater-based surveillance since gut colonization by AMR bacteria usually precedes infection and disease [11], and AMR bacteria are readily shed in stool, with additional microbiota such as from the skin, oral and upper respiratory tract also contributing to waste streams (e.g. toilets, showers, sinks). Genotypic/sequencing-based approaches to WBE may also provide useful information on specific AMR-associated genetic determinants and high-risk clones [12] whilst presenting a means to standardize AMR detection methods such as through metagenomic-based profiling of wastewater resistomes [13, 14]. WBE has established prior utility for public health outcomes in licit/illicit drug monitoring [15] and the monitoring of polio [16] but experienced a surge in interest during the SARS-CoV-2 pandemic [17] and most recently the human monkeypox (hMPXV) virus epidemic [18] and potential polio resurgence [19].

However, WBE for AMR surveillance is relatively new in terms of method development and validation. AMR-focused WBE studies have used highly heterogenous methods, probably contributing to systematic differences in outcomes and interpretations [20]. Several studies have aimed to validate specific methodology for AMR-focused WBE such as DNA extraction [21], or quantify the effect of freeze-thawing [22] or healthcare effluents [7], but many knowledge gaps remain.

A critical component of WBE is the approach used to collect wastewater for analyses. Previous work has suggested sampling of untreated wastewater influent as optimal due to transformation of microbial and AMR gene composition during treatment [20, 23, 24]. However, the composition of influent may vary significantly over short periods due to human behaviour [25] (e.g. increased flows entering the wastewater system in the morning). To account for this, methods such as flow-proportional and composite sampling combine multiple samples automatically collected throughout a defined period. These methods require autosampling equipment and multiple site visits (i.e. setup and collection), representing a significant workload. The combination of multiple samples may also prevent discrimination of peak values as reported for SARS-CoV-2 WBE [26]. Alternatively, grab sampling entails the collection of a single volume sample at one timepoint without the need for specialized equipment or multiple site visits. This is far more convenient and facilitates sampling of locations where installing equipment is not possible. However, a single sample may not be representative of daily fluctuations and could under- or over-estimate specific taxa and AMR determinants, as well as be flooded by homogenous solids [27]. Despite this, grab sampling remains the most common wastewater sampling method used by AMR-focused WBE studies [20].

Whilst more comprehensive sampling approaches are favoured for chemical target WBE, the trade-offs between the convenience of grab sampling and temporal coverage of composite sampling has not been previously explored for WBE-based AMR surveillance. We therefore conducted a high-resolution temporal study of wastewater influent comparing taxonomic and resistome profiles generated from contemporaneous hourly grab and 24 h composite sampling. Additionally, we characterized changes in influent composition in relation to metadata such as time-of-day, flow rate and nutrient concentrations. We aimed to highlight limitations of each method and recommend potential use cases.

## Methods

### Wastewater sampling

**Fig. 1.**
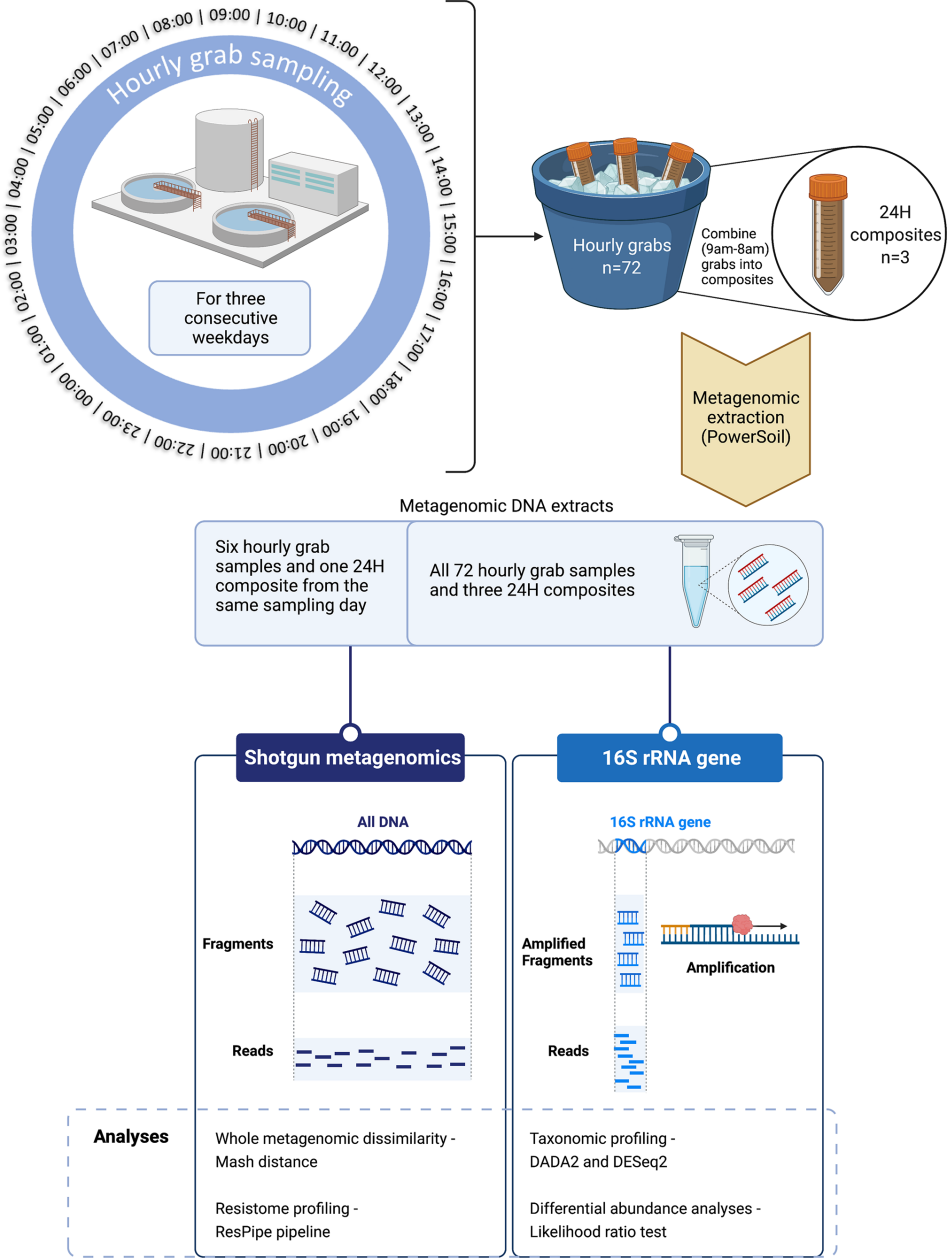
Schematic overview of study methods and analyses.

Wastewater influent was sampled from the crude inlet (post-mechanical screening out of large solids) of a large UK-based (South East England) wastewater treatment work (WWTW) with population equivalent 223 435 and consented flow of 50 985 m^3^ day^–1^. The sampled wastewater had undergone no biological or chemical WWTW treatment processes at the point of collection. The WWTW received urban wastewater and healthcare effluent from multiple sites including two large hospitals (>1 000 beds). Hourly influent grab samples (*n*=72) were collected via an ice-filled autosampler (Hach AS950) over three consecutive dry-weather weekdays [14–16 May 2019 (Tues–Thurs]) (Fig. 1). The line into the autosampler was positioned to sample mid-stream flow to avoid stagnant material, with programming to pump 400 ml of influent at an hourly interval. Each 400 ml grab was collected into a separate clean sampling bottle to facilitate processing as both grab/composite as well as visual inspection of successful sampling (i.e. no reduced volumes caused by blockages in the sampling line). Grab samples were collected every 24 h, stored on ice and processed within 3 h of collection. The autosampler was packed twice daily (morning/evening) with ice to keep samples cool before collection. A subset of hourly samples (*n*=18, 4 h interval) underwent nutrient analyses (UKCEH) as previously described [28]. In brief, this included spectrophotometric quantification, colorimetry via a Seal AutoAnalyzer 3 (Seal Analytical), thermal oxidation via a Thermalox analyser (Analytical Sciences) and ion chromatography via a Dionex AS50 (Thermo Fisher Scientific). Daily 24 h composites (*n*=3; 9 am to 8 am) were prepared by combining 100 ml from each respective grab after thorough mixing. Aliquots (100 ml) of all samples were then pelleted (5 300 r.p.m., 10 min, 4 °C) and stored at −80 °C before metagenomic extraction using the DNeasy PowerSoil kit following the manufacturer’s specifications (Qiagen), with a uniform 250 mg input for all samples. Quantification and quality of DNA extracts were confirmed using a Qubit Fluorometer and NanoDrop spectrophotometer respectively. All sampling and sample processing was conducted following cold chain principles, with storage on ice or refrigeration during sampling, transport and processing.

### Sequencing

All samples (*n*=75) underwent 16S rRNA gene sequencing using 515 F-806R primers on the MiSeq platform (Illumina) as previously described (modified Earth Microbiome Project protocol) [29] generating 250 bp paired-end reads. This was conducted using a 500-cycle V2 flowcell to achieve a depth of ~156 250 reads (~0.1 Gb) per sample. Two grab samples (2/72; timepoints 4 : 00 and 14 : 00) were excluded from analyses due to low sequencing quality. A subset of samples from day 1 [*n*=7; six grab samples (4 h sampling interval), one 24 h composite sample] also underwent metagenomic sequencing on the NovaSeq6000 at the Wellcome Trust Centre for Human Genetics, generating 150 bp paired-end reads. This was conducted to a depth of ~75 million reads (~23 Gb) per sample based on previous deep sequencing and rarefaction analyses demonstrating this as the minimum depth required to capture most AMR gene diversity in samples from the same WWTW [30].

### Computational methods

Taxonomic processing of 16S rRNA gene sequence data was conducted using DADA2 v1.16 [31] as previously described [29]. Briefly, Illumina demultiplexed 16S rRNA gene sequences underwent DADA2 workflows to quality filter, merge, denoise and assign taxonomy using silva v138.1 as the training database [32]. Nomenclature is provided according to both silva and the International Code of Nomenclature of Prokaryotes [33] where not updated by silva. The resulting amplicon sequence variant (ASV) tables were processed using PhyloSeq v1.38.0 [34] and DESeq2 v1.34.0 [35] in R v4.1.2. DESeq2 was used to address heteroscedasticity of count data via local model fitting (after visual inspection of dispersion estimates to determine the best-fitting model) and the alternative estimation of size factor method which excludes zero counts, as per recommendations for zero-inflated metagenomic count data [36]. Principal component analyses (PCAs) were conducted on variance-stabilized counts using the R packages stats v4.1.2, factoextra v1.0.7 [37], FactoMineR v2.4 [38] and vegan v2.6.2 [39] for ordination, hierarchical clustering and univariable fitting of environmental variables. Nutrient concentrations were normalized to flow rate before fitting. Significant abundance differences were assessed using a DESeq2 likelihood ratio test with a reduced design formula focused on sampling time.

Metagenomic sequencing data were processed through ResPipe [30] v1.4.0 for AMR genotyping, and Mash v2.3 for comparing overall similarity via metagenome distance estimation [40] (21 mers, sketch size=10 000, minimum abundance threshold=10 k-mers]. ResPipe outputs lateral coverage as the proportion of an AMR gene covered by at least a single base of mapped reads. A higher lateral coverage reflects more complete mapping of a gene and increased confidence in true presence, with a score of 1 representing complete capture. Low lateral coverage reflects either low abundance (i.e. few reads to map to the reference gene) or false-positives where conserved regions from different AMR determinants are erroneously mapped. The ResPipe output of AMR gene families (AGFs) was utilized to consolidate significant gene-level diversity and facilitate visualization of resistance profiles. AGF lateral coverage underwent column- and row-wise hierarchical clustering using pheatmap v1.0.12 [41] (cutree_rows=3) which produced three groups representing predominantly high or variable lateral coverage, and those with intermittently zero lateral coverage across samples (see Results). These categories represent relative confidence in the true detection of a specific AGF and provide insight into temporal variability in the context of the sampling method employed. For example, high lateral coverage of an AGF in both 24 h composites and individual grab samples would indicate temporally stable presence of that AGF. Conversely, variable or intermittently zero lateral coverage may indicate the AGF undergoes temporal flux.

## Results

### Taxonomic variation

**Fig. 2.**
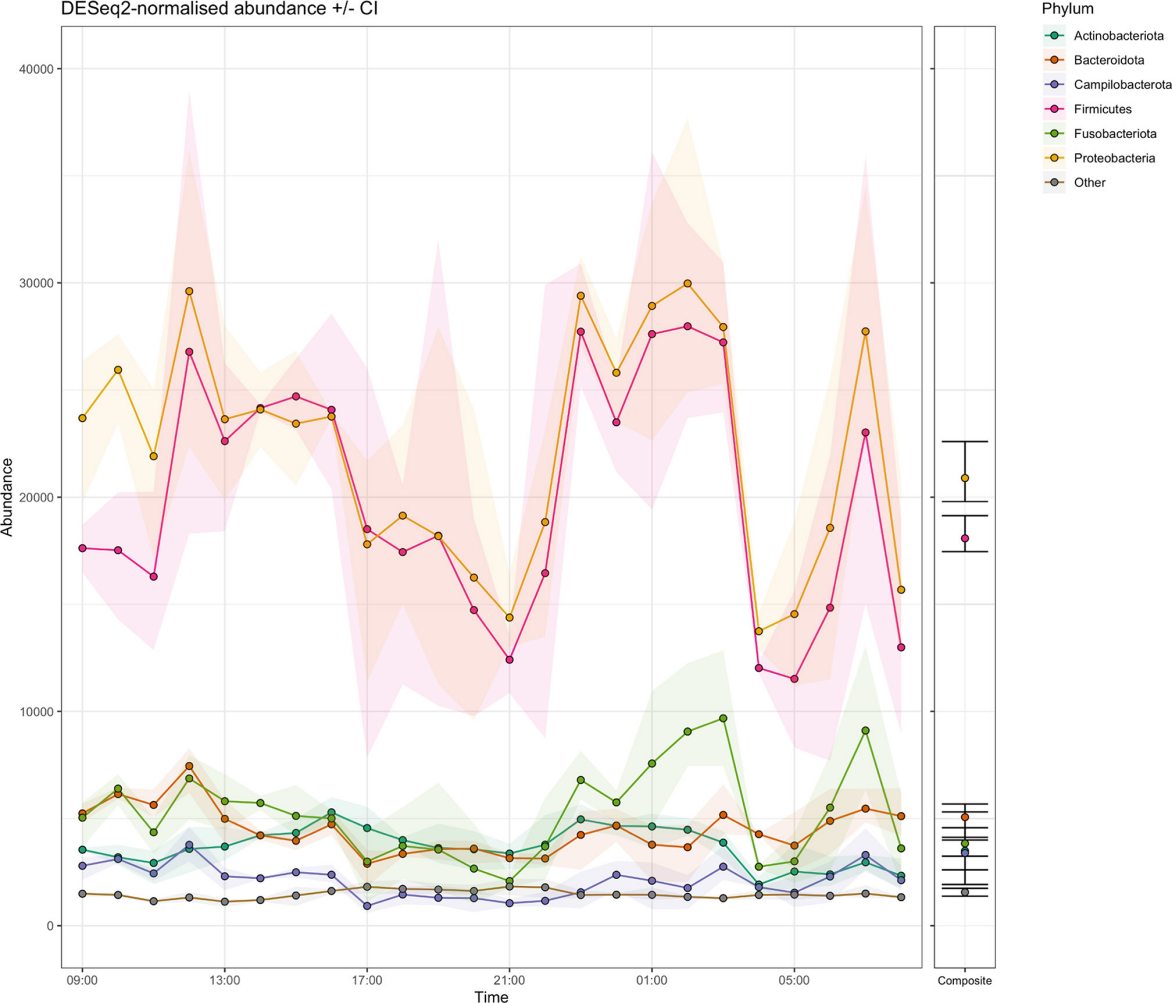
Temporal taxonomic abundance fluctuations. Each point represents the mean DESeq2-normalized abundance of phyla (coloured) across the three sampling days for hourly grab samples (left) and 24 h composites (right). Shaded areas (grab) or intervals (composite) convey 95 % confidence intervals.

**Fig. 3.**
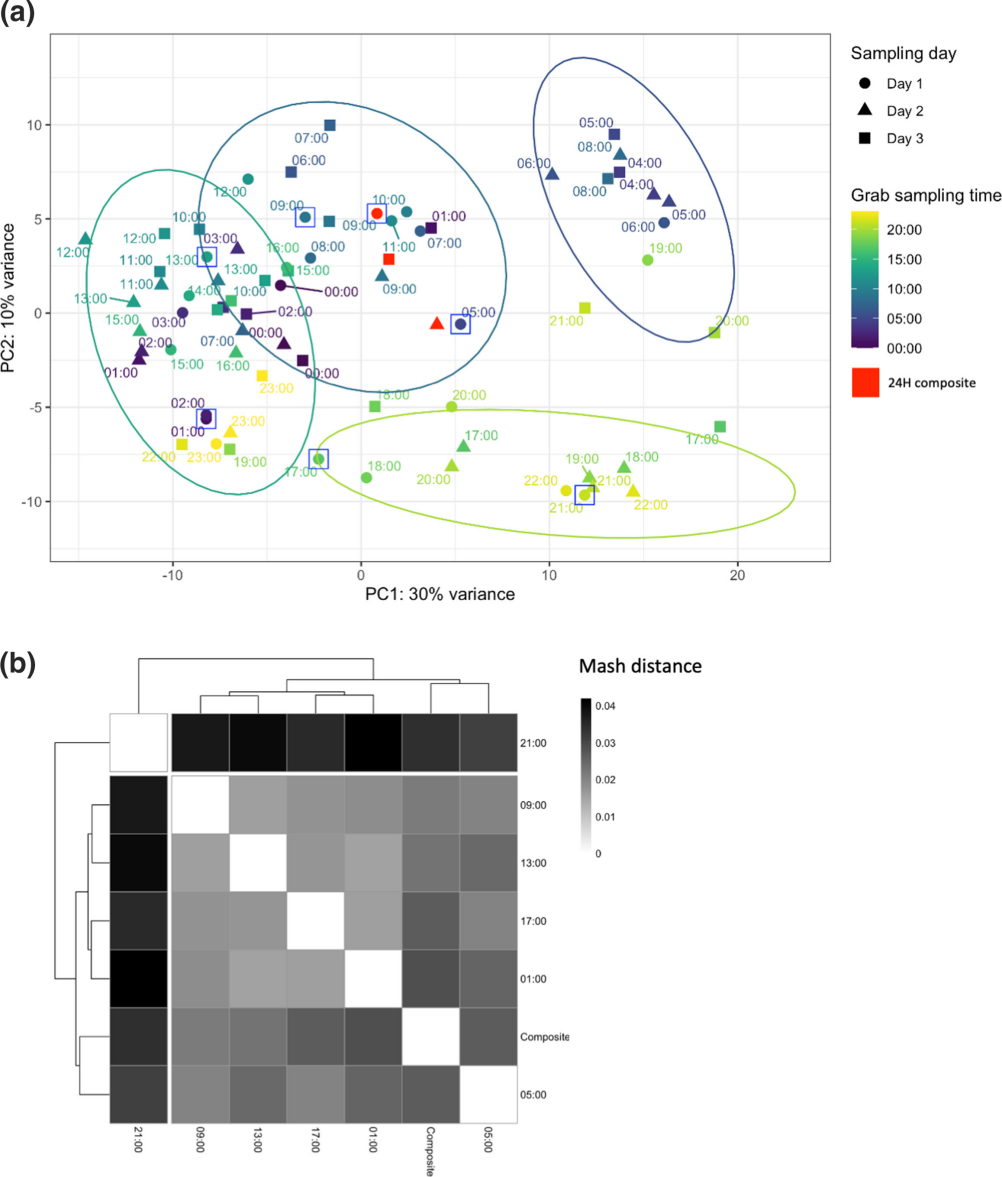
Taxonomic and metagenomic differences between samples. (**a**) Principal component analysis of taxonomic profiles for single timepoint grab samples (time of sampling labelled/coloured) and composite samples (red). Sampling day and hierarchical clusters are represented by point shape and ellipses respectively. Points highlighted by squares underwent additional shotgun sequencing. (**b**) Heatmap of Mash distance between a subset of samples from the same day (a: squares). Higher scores (darker shading) represent increased dissimilarity in pairwise comparison.

Normalized read abundances of specific phyla varied significantly across the three 24 h periods but followed a diurnal pattern with peaks at 12 : 00, 23 : 00–03 : 00 and 07 : 00 (Figs 2 and S1), most notably for *Firmicutes* (* Bacillota *) and *Proteobacteria* (* Pseudomonadota *). Remaining phyla were less abundant and more consistent across the 24 h periods but also appeared to follow a similar pattern. One grab sample (Day 2, 12 : 00) did not detect *Actinobacteria* (* Actinomycetota *), which were otherwise ubiquitous. The 24 h composites were similar for each day and appeared to approximately reflect the mean of phylum abundances across the hourly grab samples, capturing all phyla represented (Fig. S1). Assessing model fit via the likelihood-ratio test confirmed significant abundance differences attributable to grab sampling time for 77 classifiable ASVs (adjusted *P*-value<0.05, 55/77 <0.01) (Fig. S2). A batch effect was also identified between all grab samples collected on day 1 versus day 3 although this was relatively minimal as only 3/77 ASVs underwent log_2_-fold changes between these timepoints. Most ASVs belonged to *Proteobacteria* (* Pseudomonadota *) (24/77), *Firmicutes* (* Bacillota *) (22/77) and *Bacteroidota* (17/77), with the rest classified as *Actinobacteria* (* Actinomycetota *) (6/77), *Campylobacteriota* (* Campylobacterota *) (3/77), *Fusobacteriota* (2/77), *Acidobacteriota* (2/77) and *Chloroflexi* (* Chloroflexota *) (1/77). Accordingly, PCA of 16S rRNA gene-based profiles demonstrated association between time of grab sampling and taxonomic variation (Fig. 3a). Hierarchical clustering on principal components identified four main clusters with most separation for grab samples taken between 04 : 00–08 : 00 and 17 : 00–22 : 00. Consistent with the regular daily pattern across the three sampling days (Figs 2 and S1), samples taken at the same time-of-day clustered together, regardless of the day they were collected. Remaining sampling times were represented by two partially overlapping clusters. Notably, 24 h composite samples demonstrated comparably low taxonomic variability and nested centrally, surrounded by the hourly grab samples.

Mash distance of full metagenomic content for a subset of seven samples from sampling day 1 showed the same clustering pattern by PCA on 16S rRNA gene data alone (Fig. 3b). Grab samples taken at 21 : 00 and 05 : 00 were most dissimilar, corresponding to the 16S rRNA gene-based clustering around 17 : 00–22 : 00 and 04 : 00–08 : 00 respectively. The remaining grab samples were less dissimilar and congruent with the two partially overlapping clusters. As for PCA, the 24 h composite was nested between grab samples but did not exactly replicate the magnitude of variance from 16S rRNA gene data alone (e.g. composite clustered closest to 01 : 00 based on Mash distance but to 09 : 00 for 16S rRNA gene data). This probably reflects the higher resolution afforded by metagenomic Mash distance over targeted 16S rRNA gene sequencing methods.

Considering environmental variables, flow rate (*P*=0.001) (Fig. S3), dissolved fluoride (*P*=0.002) and dissolved ammonium (*P*=0.028) were independently significantly associated with 16S rRNA gene-based taxonomy, with a weaker association for dissolved nitrite (*P*=0.068) (Fig. S4). Raw nutrient concentration fluctuations are depicted in Fig. S5.

### Resistome variation

**Fig. 4.**
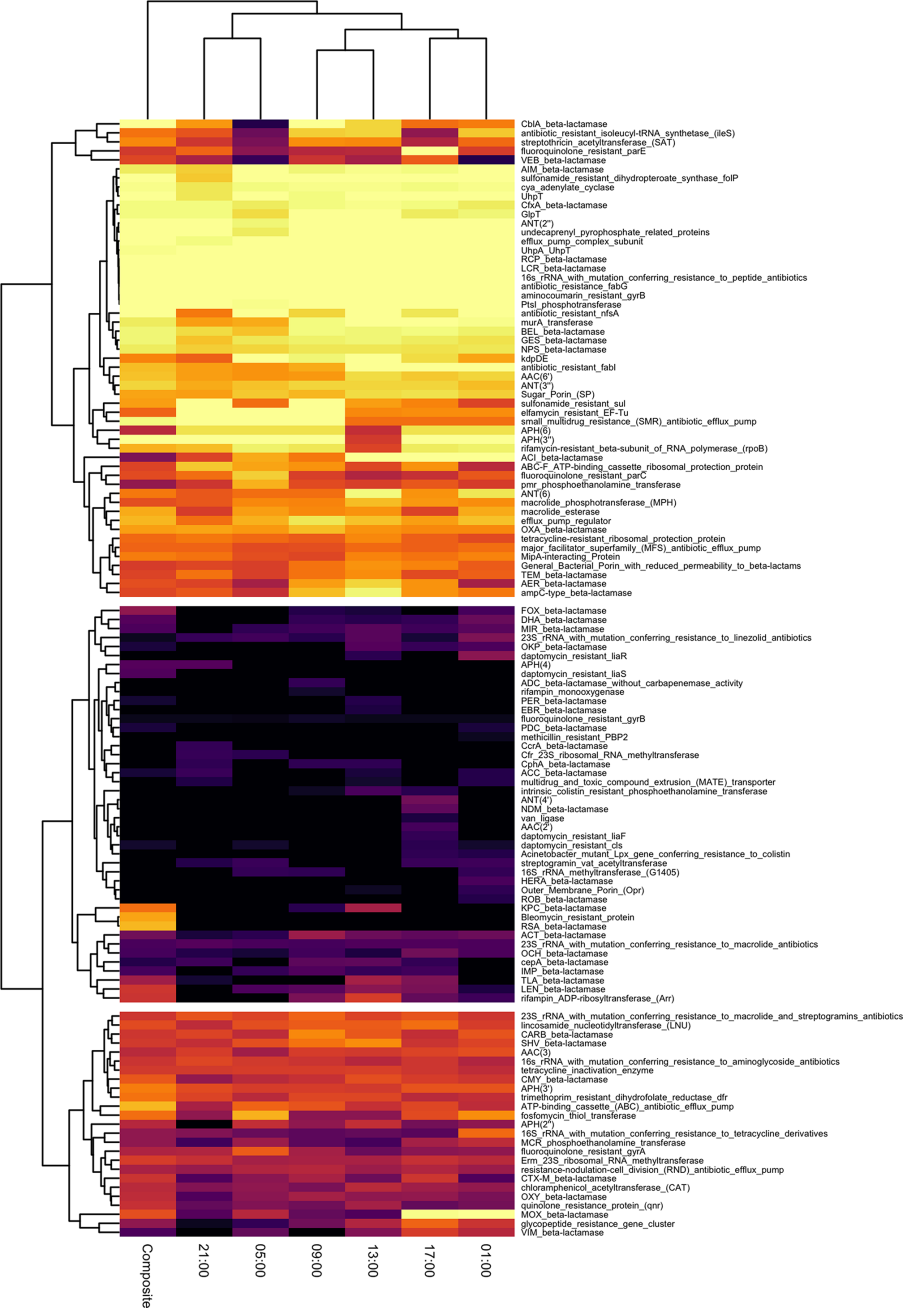
Heatmap of AMR gene family (AGF) lateral coverage across samples from the same sampling day. Row-wise hierarchical clustering divides AGFs into consistently high lateral coverage (top), intermittently 0 lateral coverage (middle) and variable lateral coverage (bottom).

From the seven samples undergoing metagenomic sequencing, in total 122 AGFs were identified at various levels of lateral coverage (i.e. mapping completeness) (Fig. 4). Of the 122 AGFs, single grab samples identified between 90/122 (74 %) and 101/122 (83 %) AGFs, while the 24 h composite identified 101/122 (83 %) AGFs. When comparing all AGFs identified in all grab samples to all AGFs identified in the 24 h composite, grab samples identified a median six [interquartile range (IQR): 5–8] AGFs not seen in the composite (total: 36 AGFs across grab samples of which 21 were uniquely identified), and the composite identified three AGFs not seen in any grab sample. However, 36/36 of grab sampling hits missed by composite hits had lateral coverage <0.5 (median: 0.19; IQR: 0.16–0.22) whereas composite hits missed by all grab samples had significantly higher lateral coverage (0.82; 0.55–0.84). Therefore, when comparing single timepoint grab sampling (i.e. considering only one grab) to 24 h composite, grab sampling probably misses more truly present AGFs and identifies more false-positives than 24 h composite sampling.

AGFs identified hierarchically clustered into three main groups based on lateral coverage across samples: predominantly high, intermittently and variably identified. In total, 53/122 (43 %) AGFs had predominantly high lateral coverage (median: 0.90; IQR: 0.74–1), 44/122 (36 %) intermittent coverage (0; 0–0.20) and 25/122 (20 %) variable coverage (0.54; 0.44–0.62). In general, variable AGFs were detected with high confidence in at least one sample. Several beta-lactamase AGFs of significant human clinical importance had lateral coverage following these groupings (Fig. S6), such as *bla*_OXA_ and *bla*_CTX-M_ families with consistently high and variable lateral coverage respectively. Notably, the carbapenemase gene families *bla*_VIM_, *bla*_KPC_ and *bla*_IMP_ were completely missed by several grab samples but identified in the composite. Conversely, the carbapenemase gene family *bla*_NDM_ was only identified in a single grab sample, and not seen in the composite.

## Discussion

In this study, we characterized the short-term temporal changes in wastewater taxonomy using 16S rRNA gene sequencing on an hourly scale over 3 days, profiled taxonomic distributions and resistome profiles using metagenomics 4 hourly throughout a single day, and concurrently compared the profiling performance of single timepoint grab sampling versus 24 h composite sampling. We find results are significantly influenced by both the sampling method used and the time of sampling for grab samples, representing an important consideration for future studies analysing wastewater.

### Temporal taxonomic changes

Taxonomic abundances fluctuated on an hourly basis and reflected a diurnal pattern which repeated for all three sampling days. *Firmicutes* (* Bacillota *) and *Proteobacteria* (* Pseudomonadota *) were the most abundant phyla overall, consistent with their classifications as dominant human gut microbiota [42]. They also constituted most ASVs (47/77; 60%) undergoing significant abundance changes. Three other human gut-associated phyla also underwent significant fluctuation [* Bacteroidota *, *Actinobacteria* (* Actinomycetota *), *Fusobacteriota *], further reflecting human faecal content in wastewater. However, whilst wastewater does reflect human microbiomes [9], its composition is also influenced by the sewer environment during conveyance to WWTWs [43], and by contributions from other wider environmental and anthropogenic sources. Accordingly, our abundance estimates for wastewater phyla are not completely consistent with estimates of *Firmicutes* (* Bacillota *) and *Bacteroidota* in direct human metagenomic studies (typically ~90 % abundance [42]), but are similar to other wastewater estimates [44–46]. *Campylobacteriota* (* Campylobacterota *), *Acidobacteriota* and *Chloroflexi* (* Chloroflexota *) also fluctuated significantly, and have been previously reported in humans, soil and wastewater respectively [47]. Interestingly, *Chloroflexi* (* Chloroflexota *) increased significantly in abundance during periods of lower flow rate, possibly indicating increased sewer community contributions [48]. We did not observe any taxonomic ‘flooding’ of grab samples whereby individual samples are uncharacteristically dominated by few taxa as reported by Reinthaler *et al*. However, two grab sample libraries of different timepoints produced low-quality data and were excluded from analyses, and may represent the stochastic collection of homogenous materials rich in amplification inhibitors such as multi-ringed polysaccharides (e.g. humic and fulvic acids) [49]. This represents a strength of compositing samples since the relative dilution of such inhibitors would aid DNA extraction/library preparation without sacrificing sensitivity as the dilutent is of interest itself.

Whilst hourly taxonomic composition fluctuated significantly, overall daily variation was relatively consistent across the three consecutive sampling days, and day of sampling did not appear to drive taxonomic variation. This was also true for 24 h composite samples which consistently approximated mean abundance of phyla from each contributing set of 24-hourly grab samples. Principal components of 24 h composites across 3 days projected closely, indicating low variation between composites, and nested centrally amongst the grab samples, suggesting representativeness. We purposefully avoided sampling on weekends and wet weather days to prevent previously described factors such as weekend household behaviour [44] and surface water runoff/infiltration [50] from impacting influent composition. Without external factors such as these, we find that sampling one weekday in our setting is representative of others in the same week, although extended longitudinal sampling would be needed to assess this in further detail.

Hierarchical clustering of principal components produced distinct clusters for grab samples taken during 04 : 00–08 : 00 or 17 : 00–22 : 00. This may further reflect changes in flow and input as these periods mostly avoid apparent flow balancing events (see below). Flow rate was also identified as significantly associated with taxonomic profiling, probably contributing both directly as a result of discharged community effluent and indirectly via carriage of sewer communities [43]. Our findings corroborate previously reported diurnal compositional fluctuations associated with flow rate related to human behaviour/household discharge patterns [25]. However, in addition to morning peaks reported by Guo *et al*., we observed additional abundance increases unrelated to household behaviour (i.e. occurring outside normal waking period) at ~01 : 00. This pattern may have been missed by Guo *et al*. due to lower resolution sampling (4-hourly) or instead reflect WWTW infrastructure differences since our sampling site utilized multiple pumping stations, balancing tanks and hydro-brakes to limit maximum flow during peak use and divert wastewater for treatment during low flow periods (e.g. overnight) (Fig. S3). We also identified significant associations to fluoride and ammonium concentrations which may suggest a role for nutrient niche-filling taxa in combination with flow as previously reported by Guo *et al*., for methanol and *Methylophilaceae *; however, the profiling of metabolism-related functional genes was beyond the scope of this study which focuses on WBE methodology for population-level AMR surveillance. In addition to 16S rRNA gene-based taxonomic assessments, metagenomic evaluation of a subset of samples showed similar clustering by sampling time, indicating that this also impacts on the capacity to detect important functional features in addition to taxonomic variation, including AMR markers. WWTW and sewer infrastructure and approaches to wastewater management are therefore important factors to consider when interpretating results, but are often overlooked [20].

### Resistome profiling

The 122 AGFs identified from the metagenomic subset of same-day samples (09 : 00, 13 : 00, 17 : 00, 21 : 00, 01 : 00, 05 : 00, 24 h composite) divided into three groups of consistent, variable and intermittent detection. When identified with lateral coverage=1 (high confidence in true presence [30]) in more than one sample, AGFs were generally also captured at one or high lateral coverage by most other samples (i.e. consistent detection), as seen for 53/122 (43 %) AGFs. This suggests parts of the resistome remain consistently detectable throughout the day and can be identified with confidence at variable timepoints; these are probably highly prevalent AMR determinants circulating in the community and/or wastewater. Variable AGFs [25/122 (20 %)] were predominantly detected with high confidence in at least one sample (e.g. MOX beta-lactamases, fosfomycin thiol transferases), and therefore are probably truly present but undergo temporal flux within a day. Thus, these probably represent circulating AMR genes or AGFs of intermediate prevalence whose identification is somewhat temporally dependent but identifiable with reasonable confidence at varied timepoints. Lastly, detection of intermittent AGFs [44/122 (36 %)] was highly time dependent, suggesting low prevalence and/or transient AMR determinants. Detecting these may rely on sampling during or temporally close to shedding events as hypothesized for SARS-CoV-2 [26, 44]. However, the low lateral coverage seen for most intermittent AGFs may alternatively represent mapping artefacts; in this case, the AGFs may not be truly present.

While grab sampling identified several more AGFs in total across all six individual timepoints, the 24 h composite more consistently identified AGFs with high lateral coverage. Almost all AGFs identified by grab samples and missed by the composite had low lateral coverage and were potentially false-positives or transiently present. Conversely, composite sampling identified AGFs likely to be truly present but missed by all grab samples. It is important to note that the 24 h composite was derived from all hourly grab samples over 24 h and not just the six timepoints which underwent metagenomic shotgun sequencing. Therefore, when an AGF such as *bla*_KPC_ is missed by most or all the six grab samples but captured with high lateral coverage in the composite, we can assume the AGF is present in other timepoints comprising the composite. If relying on grab sampling only (i.e. a single grab sample), the broader picture may be misrepresented by temporal fluctuation since a single timepoint grab only represents a narrow snapshot. For example, *bla*_VIM_, *bla*_IMP_ and *bla*_KPC_ beta-lactamases were all detected in the grab sample taken at 13 : 00, whereas all were absent in the grab sample at 21 : 00 and absent from at least one other timepoint. In this respect, the 24 h composite is more representative, capturing the intermittently detectable VIM, IMP and KPC carbapenemase families. Therefore, 24 h composites appear more reliable for detecting variable and intermittently detectable AGFs (moderate to low prevalence in community/wastewater) subject to temporal flux which may be missed by a single timepoint grab depending on sampling time.

However, sensitivity of detection also depends on sequencing depth and sample complexity [30] which may explain why certain single grab samples identified AGFs missed in composite samples. For example, *bla*_NDM_ was identified in one grab sample but completely missed in all other grabs and the 24 h composite. This may represent extremely rare circulation (consistent with local clinical prevalence [51]) which is captured by chance sampling within the timeframe of a shedding event from colonized individuals. Since this single timepoint grab sample and others are effectively diluted in the composite, sensitivity to detect low-prevalence, infrequently shed AGFs is reduced in the composite.

Further comparison to local clinical phenotypic AMR prevalence data [52] shows potential agreement between these AMR rates and lateral coverage of wastewater AGFs whereby higher rates of third-generation cephalosporin resistance are reflected by consistent detection of *bla*_CTX-M_ genes across all samples. Similarly, low clinical carbapenem resistance mirrors the intermittent low-lateral coverage detection of VIM, KPC, IMP, NDM and OXA-48-like carbapenemase genes. More work is required to link the level of circulation of AMR in a population (both clinically presenting and asymptomatic carriage) to its prevalence in wastewater to confirm the value of WBE-based AMR surveillance for public health systems. However, our findings highlight how the sampling method utilized needs careful consideration in the context of study objectives. For instance, grab sampling may be more appropriate for investigating peak values to avoid dilution in 24 h composites, but grab sampling must contend with temporal variation, flooding by homogenous materials or potential PCR inhibitors, and the stochastic nature of detecting rare targets dependent on individuals shedding in the sewershed – further compounded by population movement. Conversely, 24 h composites provide better reliability of qualitative target detection when considering temporal variation but may not capture rare targets of interest due to dilution, and so may be better suited to profiling AMR with higher prevalence of circulation.

### Limitations

Resource limitations meant that we were only able to use 16S rRNA gene sequencing to characterize the 72 hourly grab samples collected; shotgun metagenomics enables a much higher resolution and robust characterization of genetic features [53, 54]. 16S rRNA gene sequencing may also be prone to amplification biases and normalization issues. To mitigate these issues, we undertook metagenomic sequencing of a subset of samples which supported our 16S rRNA gene-based conclusions and enabled us to additionally evaluate the impact of sampling approach on resistome profiles. We also employed DESeq2 internal normalization and variance stabilizing transformation to avoid heteroscedasticity introduced by relative abundance or rarefaction methods. The sensitivity of metagenomic sequencing to accurately profile species and AMR genes in samples is dependent on sequencing depth [30]; we sequenced at an optimized depth previously determined by ultradeep sequencing of wastewater from the same WWTW as our study. We were also not resourced to produce true replicates of each timepoint grab (i.e. discrete grab samples taken at the same timepoint) which may show differences since samples were relatively small volumes collected from a large-volume system. However, our pseudo-replicates of timepoint (i.e. samples taken at the same time but different day) show low variation in contrast to comparing between timepoints, which suggests variability of discrete grabs at the same time and day would be even lower. Our time-proportional sampling method may over-represent the composition of samples collected at low flow in the 24 h composite samples due to consistent volume collection. We did not have access to flow-proportional equipment to mitigate this, but we show that time-proportional 24 h composites are representative which would probably only be improved by flow-proportional methods. We considered nutrient concentration and other environmental factors such as antibiotic concentrations would be useful to better understand the attributes of temporal change, but these analyses were beyond the scope of this study focused on whether the temporal changes are of importance to WBE. Our study was carried out in a single setting and represents sampling over 3 days of a single week only; the findings may therefore not be fully generalizable, but they nevertheless clearly highlight the relevance of considering the impact of sampling approach on results and their interpretation. The differences we observed may not be as drastic for a smaller WWTW but are probably present nonetheless as a function of flow fluctuation on microbial composition. Directly investigating the wastewater–clinical AMR overlap was beyond the scope of this study, but we did observe potential associations with local clinical phenotypic AMR prevalence data – future studies directly investigating the overlap in detail are warranted.

## Conclusion

The composition of wastewater influent undergoes significant taxonomic and resistome changes on short timescales which may affect interpretation of results based on sampling strategy. Grab sampling at a single timepoint generated significant differences in taxonomic profile which clustered based on sampling time, driven in part by human behaviour and flow rate. Although single grab sampling had greater sensitivity for potential rare/transient AMR determinants, resistome profiles were temporally variable and might not be generalizable beyond that timepoint. Additionally, many AMR determinants detected by grab sampling but missed by 24 h composites were probable false-positives. The 24 h composites reflected overall daily taxonomic composition and more reliably captured AMR determinants present throughout the day or completely missed by grab sampling.

Consequently, we recommend 24 h composite sampling over single timepoint grab samples where feasible for reliable qualitative profiling of most AMR determinants. If grab sampling is undertaken for convenience, the assessment of peak values or identification of rare circulating determinants, consideration should be given to the fact that it may misrepresent taxonomic and AGF abundances due to temporal flux. Future work identifying an optimal sampling time for grab sampling might be useful but challenging due to extensive differences in relevant factors in different settings such as sampling site size, population and infrastructure. Passive sampling methods may also represent an effective alternative to traditional grab or composite sampling, and have shown potential in monitoring SARS-CoV-2 [55]. Validation and optimization of WBE methods for AMR surveillance is vital for its development as a robust surveillance approach; context-specific validation for methods selected could be useful to ensure that results and interpretation are robust.

## Supplementary Data

Supplementary material 1

Supplementary material 2
