# Supplementary material for: High-resolution characterization of short-term temporal variability in the taxonomic and resistome composition of wastewater influent
Source: Microb Genom. 2023 May 5;9(5):mgen000983. doi: 10.1099/mgen.0.000983 (PMC10272859; doi:10.1099/mgen.0.000983)
Supplement: Supplementary material 1 [file mgen-9-983-s001.pdf]

Appendix

Figure S1: Temporal taxonomic abundance fluctuations faceted by sampling day. DESeq2-normalised abundance of Phyla (coloured) across the three sampling days for hourly grab samples (left) and 24H composites (right).

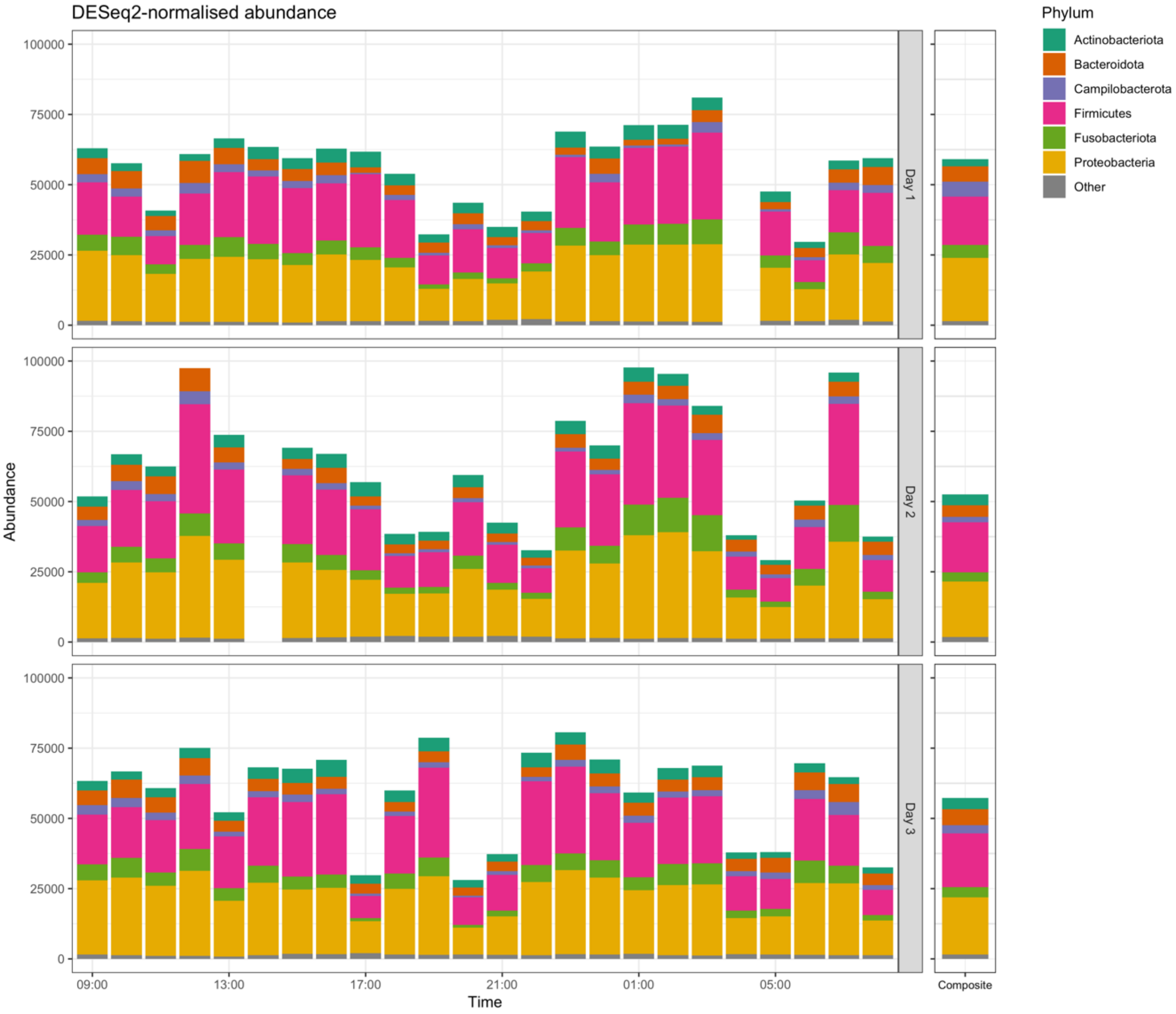

Figure S2: Log 2-fold changes in abundance for ASVs undergoing significant abundance changes across grab samples. Each row represents an ASV identified as significant by likelihood ratio testing in at least one pairwise comparison between samples (columns). Maximum threshold of change was set at 2 and -2 to aid visualisation of significant abundance differences (i.e. values at 2 and -2 may represent >2 and <-2 respectively). Phylum-level classification of ASVs is annotated by colour (left).

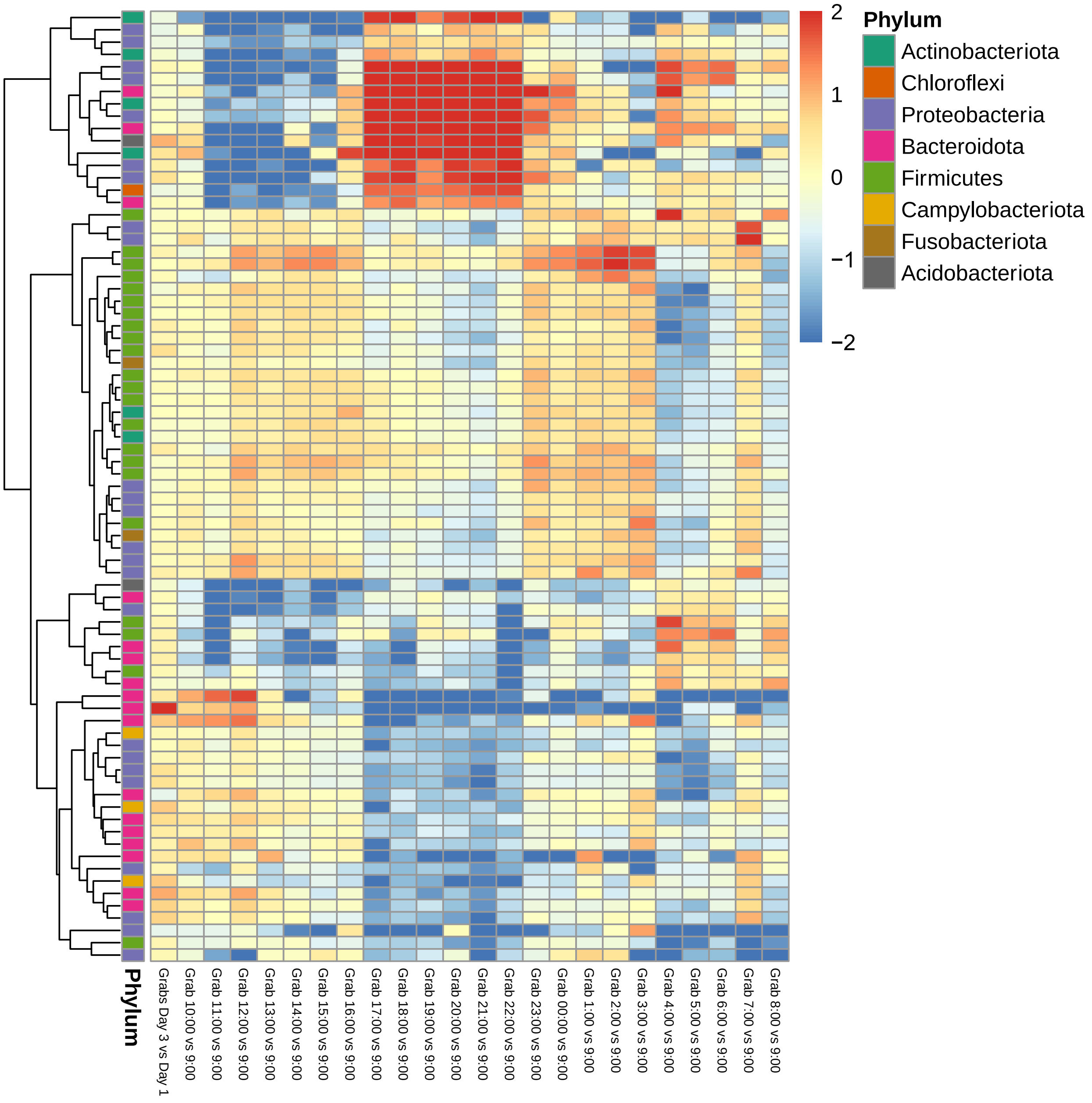

Figure S3: Mean flow rate of influent across sampled three days
Ribbons (grey) represent 95% confident intervals for the mean flow rate measured at 15 minute intervals.

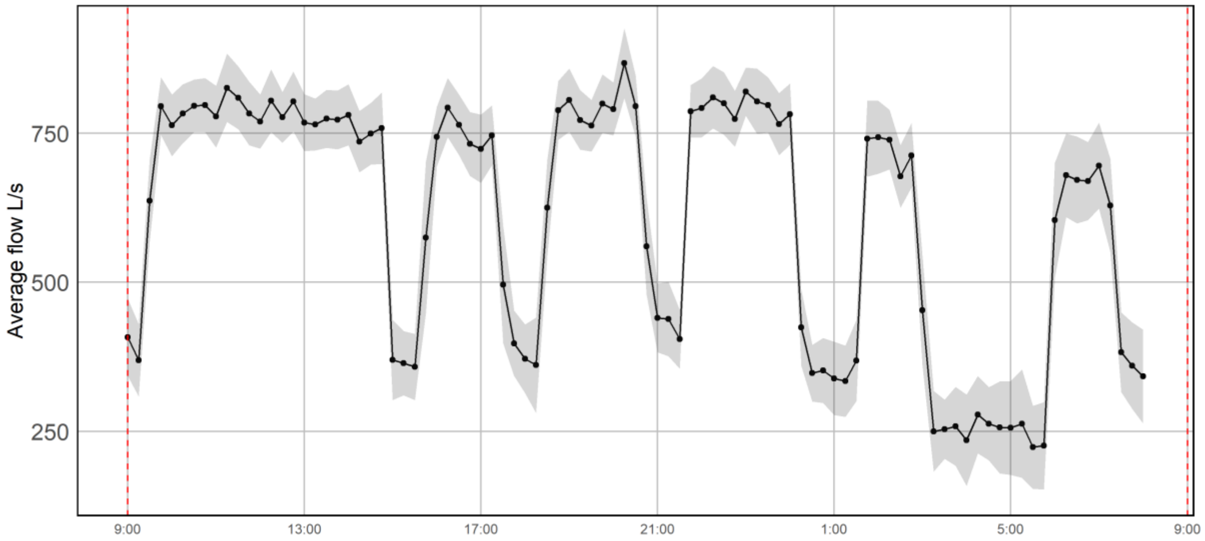

Figure S4: Principal component analysis of taxonomic profiles across samples with fitted environmental variables. Arrows are labelled with environmental variables where direction and magnitude indicate explanation of sample variance.

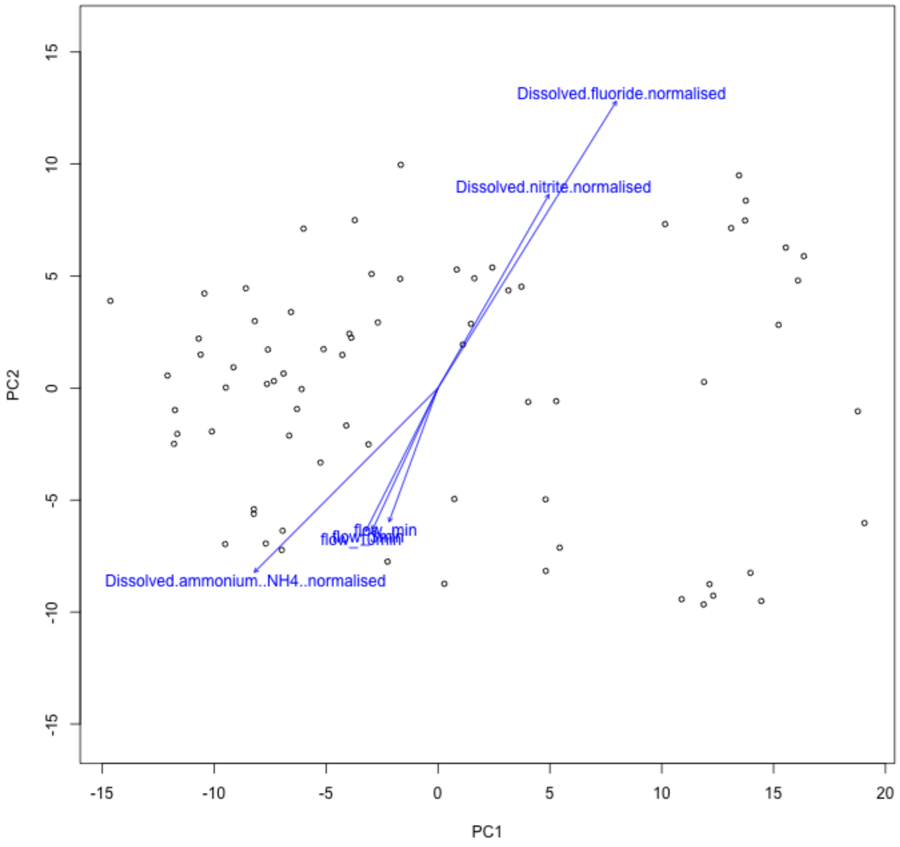

Figure S5: Raw nutrient measurements for single timepoint grab samples

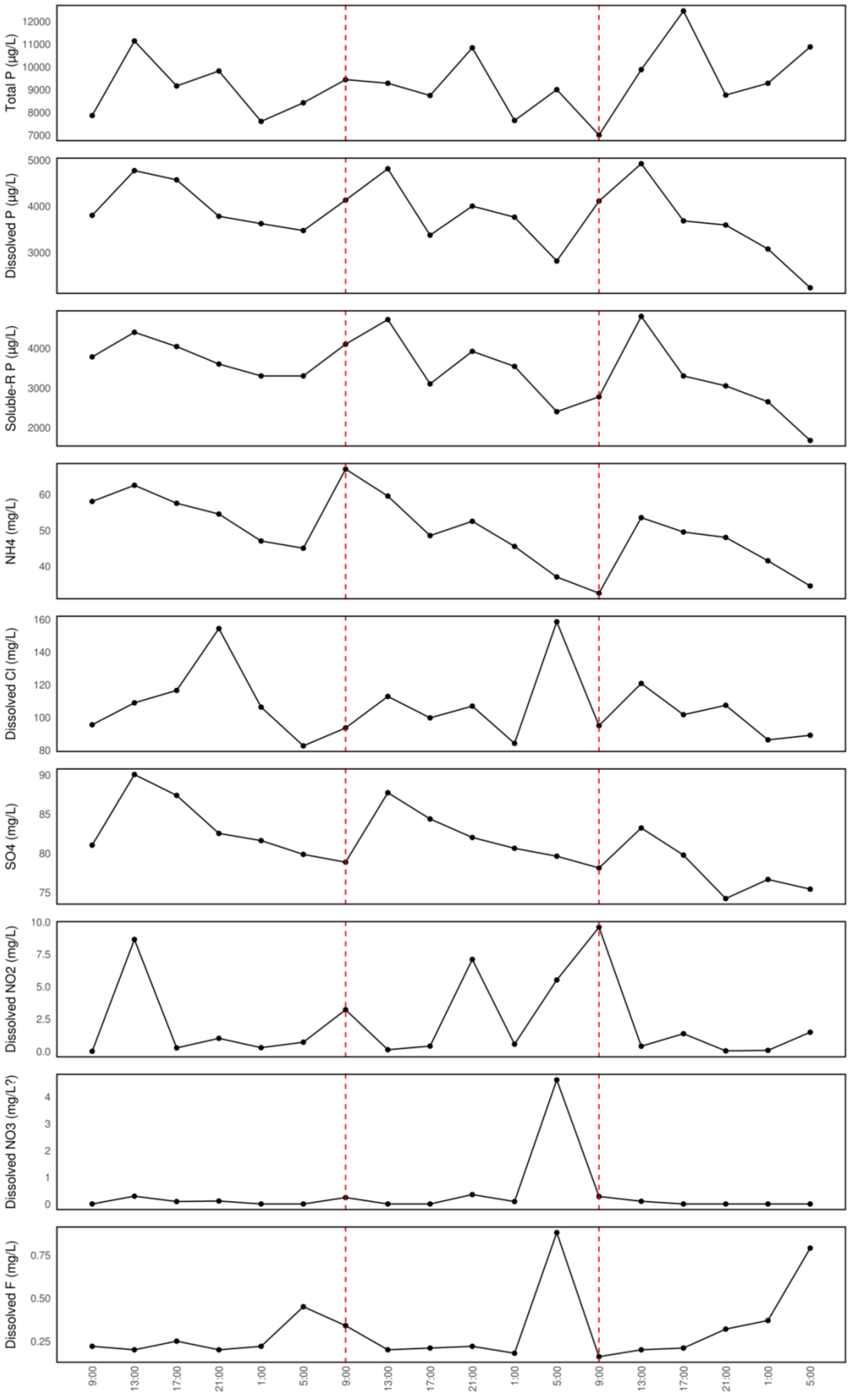

Figure S6: Heatmap of selected AMR gene family (AGF) lateral coverage across samples from the same sampling day. Subset of significant clinically relevant AGFs of particular interest to public health surveillance.

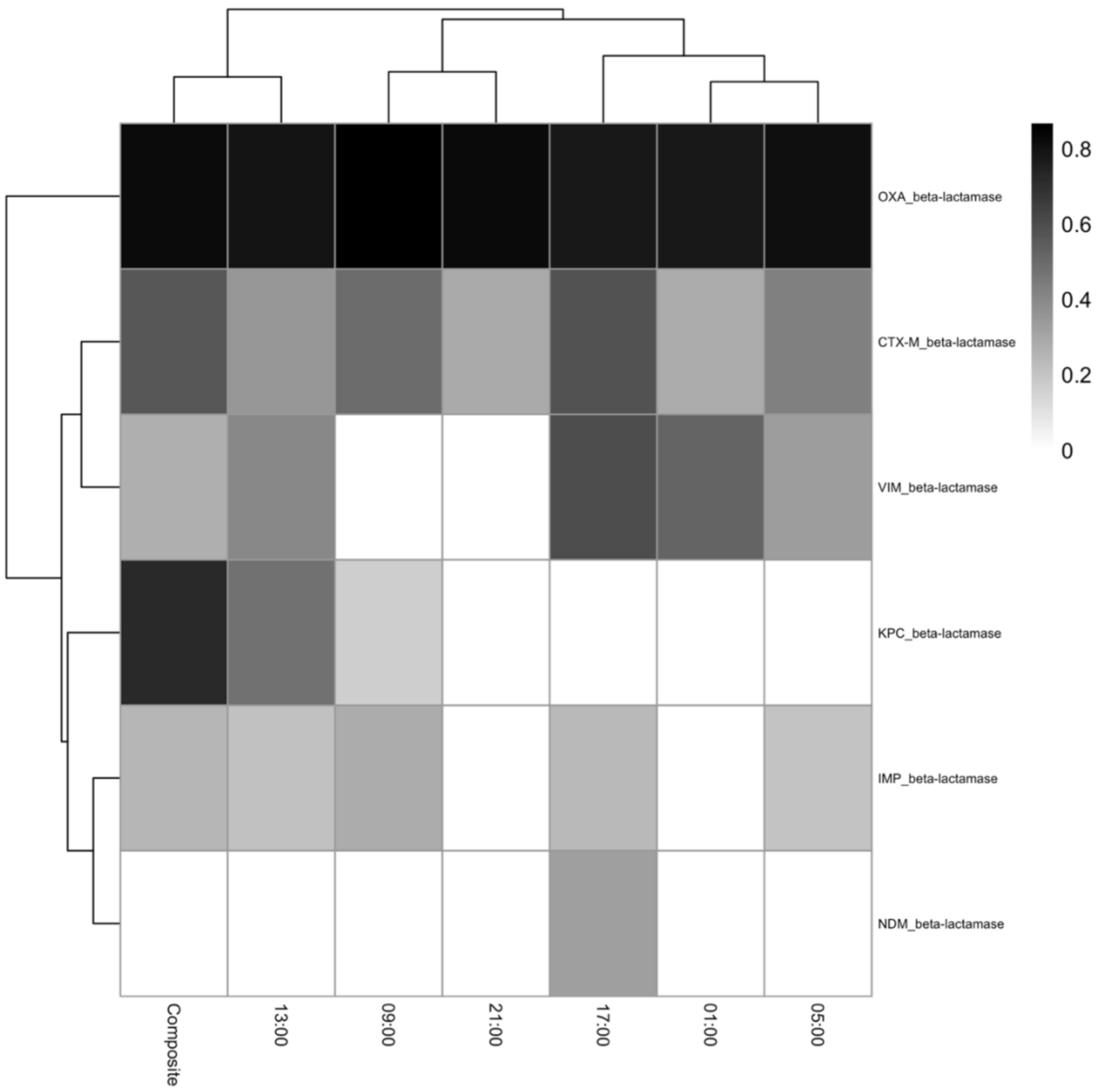

Complete Author contributions (as per CRediT; Contributor Roles Taxonomy)

Conceptualization

KK Chau, DS Read, N Stoesser, AS Walker

Methodology

KK Chau, DS Read, N Stoesser

Software

KK Chau, DS Read, N Stoesser, T Goodall

Validation

KK Chau, N Stoesser

Formal Analysis

KK Chau, N Stoesser

Investigation

KK Chau, N Stoesser, T Goodall, M Bowes

Resources

KK Chau, DS Read, K Easterbrook, H Brett, J Hughes

Data curation

KK Chau, N Stoesser, T Goodall, K Easterbrook

Writing – original draft

KK Chau, N Stoesser

Writing – review & editing

KK Chau, N Stoesser, DS Read, AS Walker, T Goodall

Visualization

KK Chau, N Stoesser

Supervision

KK Chau, DW Crook, DS Read, N Stoesser, AS Walker

Project administration

KK Chau, DS Read, N Stoesser

Funding acquisition

KK Chau, DW Crook, DS Read, N Stoesser, AS Walker
